# Control of high-speed jumps: the rotation and energetics of the locust (*Schistocerca gregaria*)

**DOI:** 10.1007/s00360-022-01471-4

**Published:** 2023-01-30

**Authors:** C. K. Goode, Gregory P. Sutton

**Affiliations:** School of Life Sciences, Joseph Banks Laboratories, University of Lincoln, Beevor Street, Lincoln, LN6 7DL England, UK

**Keywords:** LaMSA, Pitch, Invertebrate, Energy budget, Jumping, Biomechanics

## Abstract

Locusts (*Schistocerca gregaria*) jump using a latch mediated spring actuated system in the femur-tibia joint of their metathoracic legs. These jumps are exceptionally fast and display angular rotation immediately after take-off. In this study, we focus on the angular velocity, at take-off, of locusts ranging between 0.049 and 1.50 g to determine if and how rotation-rate scales with size. From 263 jumps recorded from 44 individuals, we found that angular velocity scales with mass^−0.33^, consistent with a hypothesis of locusts having a constant rotational kinetic energy density. Within the data from each locust, angular velocity increased proportionally with linear velocity, suggesting the two cannot be independently controlled and thus a fixed energy budget is formed at take-off. On average, the energy budget of a jump is distributed 98.7% to translational kinetic energy and gravitational potential energy, and 1.3% to rotational kinetic energy. The percentage of energy devoted to rotation was constant across all sizes of locusts and represents a very small proportion of the energy budget. This analysis suggests that smaller locusts find it harder to jump without body rotation.

**Supplementary Information:**

The online version contains supplementary material available at 10.1007/s00360-022-01471-4.

## Introduction

Locusts (*Schistocerca gregaria*) use a three-stage process to generate a jump. In the first stage, just prior to the jump, Locusts use a geometric latching system to lock their metathoracic (hind) legs in place (Heitler 1977; Divi et al. 2020). In the second stage, the large tibiae extensor muscles slowly contract to store mechanical energy in two spring-like elastic structures in the leg, the semilunar process and extensor apodeme. It can take the locust hundreds of milliseconds to load these springs (Heitler and Burrows 1977). After the energy is stored, the latch is released, and the third stage of the jump begins, where recoil of the springs then extends the legs, powerfully accelerating the animal into the air (Bennet-Clark 1975). This use of a spring/latch system to generate a powerful jump, make the locust jump a textbook (Patek and Biewener 2018; Alexander 1988; Chapman 1998) example of a latch mediated spring actuated (LaMSA) (Ilton et al. 2018; Longo et al. 2019) behaviour.

For a locust to successfully jump to a target requires the control of speed, elevation (up-down angle), and azimuth (left–right angle). Locusts control the speed of a jump by adjusting the amount of energy stored in the semi-lunar processes and extensor apodemes (Bennet-Clark 1975; Sobel 1990). The angle of elevation is determined by the position of the metathoracic legs, prior to take-off, and can be fine-tuned by adjusting the angle of the femur at the coxa body joint (Sutton and Burrows 2008). Speed and take-off elevation combined determines the distance of a jump (Bennet-Clark 1975; Sobel 1990). Azimuth is controlled, prior to and during take-off, by the position and extension of the prothoracic (front) and mesothoracic (mid) legs. Motions of the forelegs (front) can alter azimuth to a maximum of 50° to the left or right (Santer et al. 2005). Counterintuitively, while locusts with only one hind leg experience a loss in overall power produced per jump, they suffer no deficits in controlling a jump’s azimuth or elevation (Santer et al. 2005; Sutton and Burrows 2008). The trajectory of a jump consists not only of the linear vector of movement, but also the rotation (pitch, roll, and yaw) of the body at take-off.

There is not much known, however, about the control of rotation, particularly pitch, in locusts. Previous studies in other invertebrate jumpers include analysis of pitch in trap-jaw ants (*Odontomachus bauri*), which use their mandibles to propel defence and escape jumps, during which the ants body experiences rotation rates of up to 63 rev s^−1^ (Patek et al. 2006). Praying mantises (*Stagmomantis theophila*) control their pitch in air with counter rotation of their front legs, back legs, and abdomen, which reduces the momentum of their trunk, and have slower overall rotations (2–3 rev s^−1^) (Burrows et al. 2015; Sutton et al. 2016). Jumping plant lice (*Psyllidae*) jump with angular rotations of hundreds of Hz (average 336 Hz), and do not appear to use their wings to stabilise these fast rotations (Burrows 2012). Likewise, adult locusts experience a pitch rotation at take-off with rotation rates as high as 2.1 Hz being observed (Gvirsman et al. 2016; Cofer et al. 2010). The rotation in locusts was found to be produced by a positive and negative torque acting on the locust during and after take-off. The negative torque is caused by the centre of mass (COM) sitting below the plane of the thrust vector during take-off, causing head-down rotation. A positive torque is produced by flexion of the dorso-longitudinal muscles during and after take-off to counteract the head-down rotation (Baader 1990; Cofer et al. 2010). In most jumps, the sum of these torques will cause the locust to rotate head-up tail-down (for example, over 90% of these jumps rotated this way in Cofer et al. 2010). This ‘head up, thorax down’ bias is seen in almost every study of locusts jumping (and is explicitly commented upon in Bennet-Clark 1975; Sutton and Burrows 2008; Cofer et al. 2010; and Gvirsman et al. 2016). Because the energy budget for a grasshopper jump is constrained by the energy that is stored in the elastic processes of the limb, energy used for rotation has to come at a cost to energy used to generate linear velocity. This trade-off would exist in any LaMSA jumping animal. While the previous work looked at the rotation rates of the insects, none of these papers combined the linear velocity with the angular velocity to analyse the complete energy budget of a jumping animal: i.e. what percentage of the energy stored in the spring is turned into linear velocity and what percentage is turned into angular velocity, i.e., does rotation significantly affect the energy budget of a jump?

With this in mind, we aim to further investigate angular rotation (pitching) in locusts (*Schistocerca gregaria*) and determine the partitioning of energy during a jump, showing that rotation is about 1% of the energy budget. We will also investigate the quantitative relationship between size and rotation rate, showing that the proportion of translational and rotational kinetic energy remains constant across two orders of magnitude of size of locust, with smaller locusts commensurately spinning more quickly to maintain this energy partition. Consequently, determining how translational and rotational kinetic energy are portioned within the strict energy budget of a LaMSA jumper, and how size affects this budget.

## Methods

Captive-bred locusts (*Schistocerca gregaria*) ranging from first instar to adults were stored together in a terrarium kept at 30 °C with a 12/12 light/dark day cycle. The locusts were fed and watered ad libitum with polyacrylamide water gel and wheat germ. Locusts were randomly selected and weighed using an analytical balance. Body mass ranged from 0.049 to 1.5 g. Adult female locusts above 2 g were presumed gravid and were therefore returned to the terrarium. Locusts with any missing legs were also returned.

**Fig. 1:**
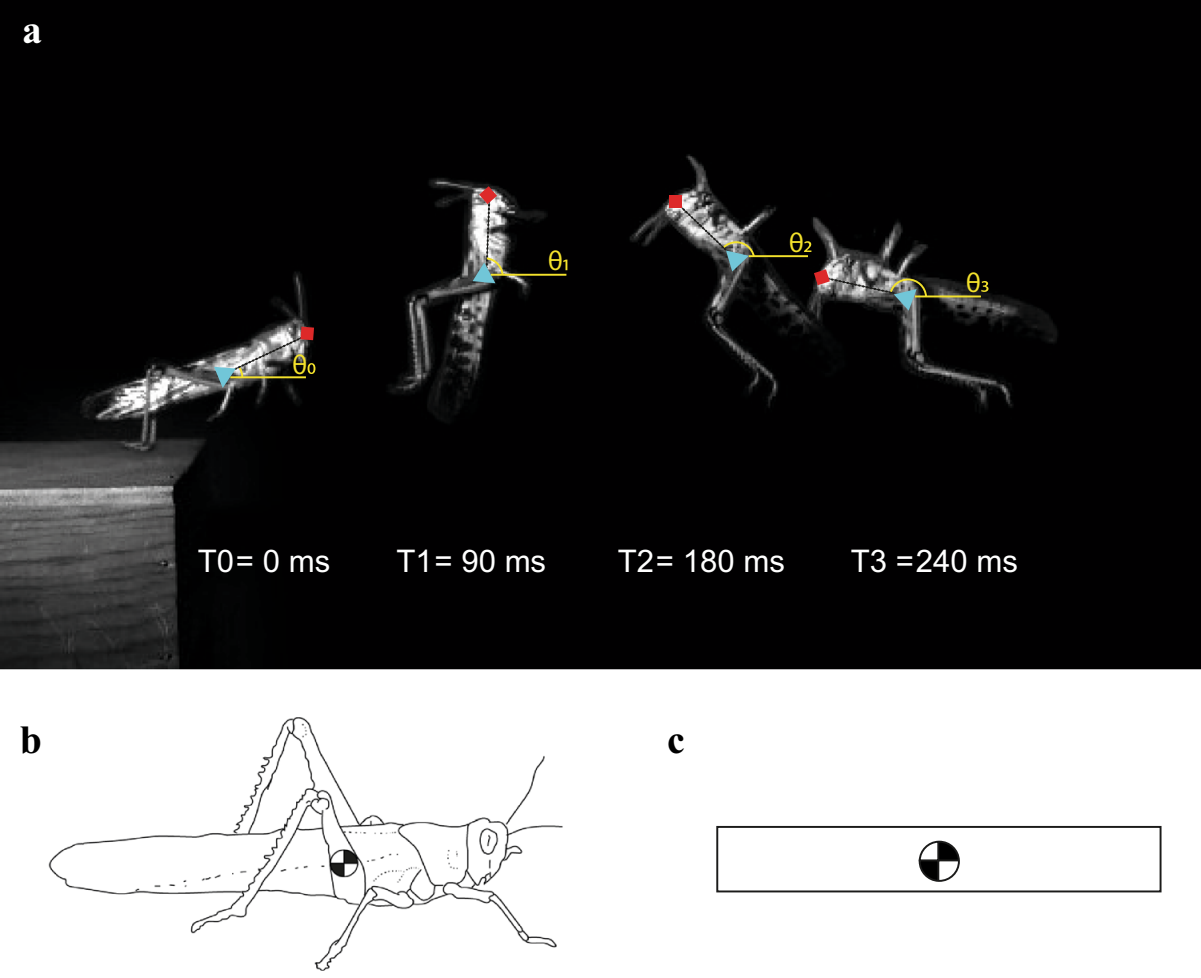
Analysis of a locust (*Schistocerca gregaria).* **a** Tracking a locust jump from take-off T0 = 0 ms to T3 = 240 ms. The position of the blue triangle centre of mass (COM), red square Head were tracked using Tracker (Open Source Physics, 2020). These were used to calculate the angle of the body (q) for each frame. **b** The centre of mass of a locust is located above the coxa of the metathoracic leg (Bennet-Clark 1975). **c** The kinematic model of a locust represents the locust as a uniform rod that rotates about its middle, where the centre of mass is located

The locusts jumps were recorded using a Photron FastCam Mini filming from a lateral perspective, orthogonal to the direction of the jump. This perspective allowed the position of the locust’s head and centre of mass (COM) to be tracked throughout the jump (Fig. 1a). Jumps with azimuth greater than 10° to the left or right quickly move out of the camera’s plane of focus and were therefore discarded.

Jumps were filmed at 1000 frames s^−1^ with an exposure time of 1 ms. A flicker free LED light was used to illuminate the jumping arena. Locusts were individually placed on a wooden podium (10.2 h × 12.7w × 5.1d (cm)), 30 cm away from their target, a horizontal bark-like textured terrarium wall. The locusts were encouraged to jump, from the podium to the wall, by quickly introducing an object, such as a paintbrush, into their visual field. Sheets of black felt were used on all other exposed areas of the arena, to remove any stimulus that might encourage the locusts to jump off target. A small filament lamp placed above the target was effective in enticing the locusts to jump toward the target.

Videos were analysed using Tracker Video Analysis and Modelling Tool (Open Source Physics, 2020). The height of the podium was used as a reference length, to measure the locust’s body length, from the head, along the dorsal line, to the most distal point of the abdomen. The (x, y) coordinates of the head and COM were used to calculate the linear velocity ($v$) (m/s) (Eq. 1), translational kinetic energy (${\mathrm{E}}_{\mathrm{T}}$) (mJ) (Eq. 2), angular rotation rate ($\dot{\theta }$) (rad/s) (Eq. 3), rotational kinetic energy (E_R_) (mJ) (Eq. 4), and gravitational energy (GPE) (m.g.h) (Eq. 5), with h being the height of the locust at take-off. Translational kinetic energy was calculated as the sum of translational kinetic energy and gravitational potential energy, to account for the different take-off heights for locusts of different sizes (Scholtz et al. 2006). The mass and length of the locust was used to calculate the moment of inertia (I) (Kg.m^2^) (Eq. 6). The equations are presented below.

### Equations

1 $$\mathrm{Linear\;Velocity }\left(v\right)\left(\mathrm{m}/\mathrm{s}\right)\quad v\,=\sqrt{{\begin{array}{c}\left(\frac{dx}{dt}\right)\end{array}}^{2}+{\begin{array}{c}\left(\frac{dy}{dt}\right)\end{array}}^{2}}$$

2 $$\mathrm{Translational \,Kinetic \,Energy }\left({\mathrm{E}}_{\mathrm{T}}\right)\left(\mathrm{mJ}\right)\quad KE=\frac{1}{2}m{v}^{2}$$

3 $$\mathrm{Angular \,Velocity }\left(\dot{\uptheta }\right)\left(\mathrm{rads/s }{\mathrm{}}^{}\right) \quad\dot{\uptheta }= \left(\frac{\mathrm{d}\theta }{dt} \right)$$

4 $$\mathrm{Rotational \,Kinetic \,Energy}\, \left({\mathrm{E}}_{\mathrm{R}}\right)\left(\mathrm{mJ}\right)\quad RE =\frac{1}{2}I{\dot{\theta } }^{2}$$

5 $$\mathrm{Gravitational \,Potential \,Energy }\,(\mathrm{GPE})\left(\mathrm{mJ}\right)\quad GPE\;=\;m.g.h$$

6 $$\mathrm{Inertia \,of \,a \,Rod \,about \,its \,centre }\,\left(I\right)(\mathrm{Kg}.{\mathrm{m}}^{2})\quad I\;=\;\frac{1}{12}{(Mass.Length}^{2})$$

Excel was used for statistical analysis. On each graph, every point represents the mean value from multiple jumps from an individual locust. Data for animal 44 is presented as the mean + / − standard deviation. For all regression tests, *p* =  < 0.05 was used as the threshold value to signify statistical significance.

After take-off insects visibly decelerate. Therefore, in this study we only measured rotation rates immediately (10 ms) after take-off and did not measure subsequent deceleration.

In total, 44 locusts were used in this experiment. Each locust was filmed jumping a minimum of 1 and a maximum of 11 times, with the exception of animal 44 which was jumped 61 times. A total of 263 videos were used in the final analysis for this study. Summaries of the entire data set are presented as means + / − standard error. Parameters for the individual 44 locusts are, unless otherwise stated, the mean for that locust from its individual jumps. To analyse inter-individual variation, we filmed 61 consecutive jumps, over a three-hour period, from animal number 44. The mean from this individual is included in our data set (for a ‘N’ of 44 locusts), but its data point is shown on each graph in yellow with black standard error bars. Over the 61 jumps, there was no significant difference in take-off velocity or take-off angular velocity showing that the animal did not experience fatigue (data not shown).

### Modelling

The centre of mass of the locust is located above the coxa joint on the abdomen (Bennet-Clark 1975). The abdomen of the locust is flexible and can be moved through contraction of the dorso-longitudinal muscles (Baader 1990). After take-off and during flight, flexion of the abdomen has been shown to create a counter torque to reduce angular rotation (Cofer et al. 2010). However, in this study we only focus on the first 10 ms of the jump after take-off, during which, angular rotation was not seen to be corrected through movements of the abdomen (see Fig. 1 for an example). Therefore, the locusts in this study were modelled as a rigid uniform rod (Fig. 1c). This model allows us to calculate the inertia (Eq. 6) of the locust using the standard equation of a uniform rod rotating about its centre (Idema 2018).

### Hypotheses

We have three mutually exclusive hypotheses for the relationship between pitch velocity and mass, each predicts a different relationship between slope and angular velocity.

Our first hypothesis, that angular velocity is invariant with mass, predicts that there will be no correlative relationship between an animal’s size and its rotation speed, thus predicting that the relationship between an animal’s mass (m) and angular velocity ($\dot{\theta }$) will have a slope that is not significantly different from zero. This hypothesis predicts that angular velocity and linear velocity would scale identically with mass, i.e., neither vary with mass.

$${\text{Hypothesis}}\,{\text{one}}:\,\mathop \theta \limits^{.} \,is\,proportional\,to\,m^{0.0} (Hyp1)$$

Our second hypothesis, that angular acceleration is invariant with mass, predicts that larger insects, which are in contact with the ground for a longer time, will be able to generate larger angular velocity. Because time in contact with the ground is proportional to leg length, this hypothesis predicts that the angular velocity will also be proportional to leg-length, i.e., there must exist a scaling exponent of 0.33 between mass and angular velocity.

$${\text{Hypothesis}}\,{\text{two}}:\,is\,proportional\,to\,m^{0.33} \,(Hyp2)$$

Our third hypothesis, that angular energy density is invariant with mass, predicts that smaller insects will generate larger angular velocities. The energy in rotation of a rotating insect is equal to $\frac{1}{2}I\dot{\theta }$ ^2^, or equivalently $\frac{1}{24}m {L}^{2}\dot{\theta }$ ^2^. Energy density equals rotational kinetic energy divided by the mass, or $\frac{1}{24}$ L^2^ $\dot{\theta }$ ^2^. For the energy density to be constant, the angular velocity ($\dot{\theta })$ must scale inversely to length (L). Length scales with mass^0.33^, and thus energy density can only be constant if angular velocity scales with mass^−0.33^. Thus, we have our third hypothesis.

$${\text{Hypothesis}}\,{\text{three}}:\,is\,proportional\,to\,m^{- 0.33} (Hyp3)$$

These hypotheses are based on the assumption that linear take-off velocity is independent of the insect’s mass, as is consistent with the mass-invariant take-off velocities often seen in LaMSA jumping insects (Ilton et al. 2018; Longo et al. 2019; Sutton et al. 2019).

## Results

### Kinematics of the jump

**Fig. 2:**
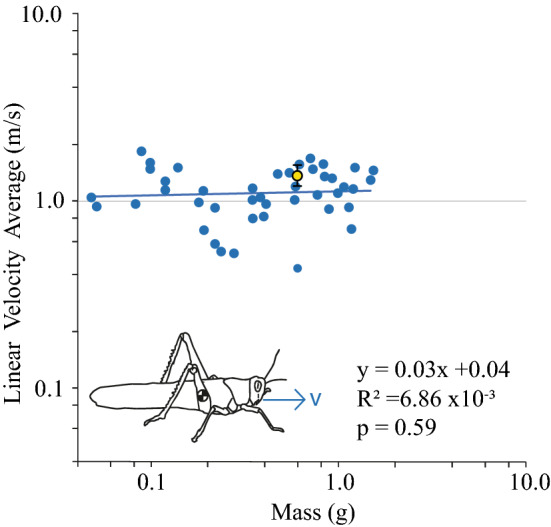
Linear velocity (v) (m/s) of a locust’s jump at take-off. Linear velocity does not vary with mass (*p* = 0.59, *R*^2^ = 6.86 × 10^−3^, Regression test). A line with a slope of y = 0x + c (shown in grey), is plotted to show the similarity in slope to our statistical trendline (blue)

Linear velocity (m/s) at take-off did not vary with the mass of a locust (Fig. 2). The average velocity for the smallest locust (0.049 g) was 1.03 m/s. The average velocity for the largest locust (1.50 g) was 1.44 m/s. Across 44 locusts, the average linear velocity ranged from 0.43 to 1.85 m/s. The absolute maximum and absolute minimum linear velocity recorded across all jumps were 1.96 and 0.25 m/s. The average velocity for animal 44 (Shown in yellow) was 1.39 m/s ± 0.18 (SD).

The correlation between average linear velocity and mass (*y* = 0.03 × + 0.04) was not significantly different (*p* > 0.05) from y = 0x + c, indictive of no correlation, (*p* = 0.59, *t* = 0.54, *N* = 44, *R*^2^ = 6.86 × 10^−3^, Statistical Regression test). This is consistent with locusts being latch-mediated spring actuated jumpers. Consequently, this result is typical for locusts (Katz and Gosline 1992), of other LaMSA systems (Ilton et al. 2018). Adult locusts are capable of generating jumps as fast as 3–4 m/s (Bennet-Clark 1975; Gabriel 1985). However, our take-off velocities were much less, around 1–2 m/s, which suggests that our jumps were submaximal and targeted at the wall of the arena.

**Fig. 3:**
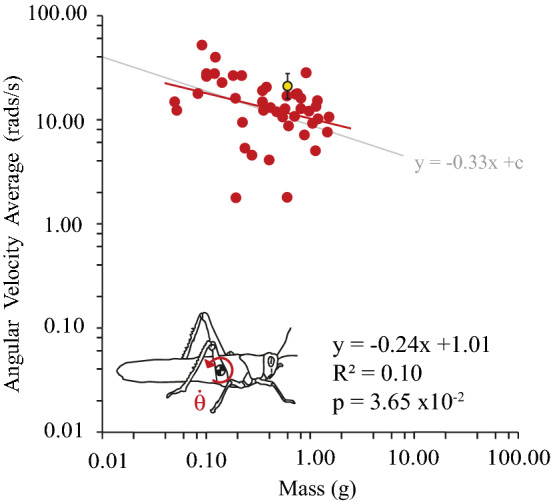
Angular velocity ($\dot{\theta }$) (rads/s) of a locust’s jump, at take-off. Average angular velocity decreases with mass^−0.33^ (*p* = 3.65 × 10^−2^, *R*^2^ = 0.10, regression test), thus the angular energy density remains constant as mass increases. A line with a slope of *y* =  − 0.33 × + c (shown in grey) is plotted to show the similarity in slope to our statistical trendline (red)

As locusts increase in size, their angular (pitch) velocity decreases (Fig. 3**)**. The average angular velocity for the smallest locust (0.049 g) was 14.86 rads/s. The average angular velocity for the largest locust (1.50 g) was 10.75 rads/s. Across 44 locusts the average angular velocity ranged from 1.75 to 51.08 rads/s. The absolute maximum and minimum angular velocity recorded across all jumps were 60. 41 and 0.11 rads/s. The average angular velocity for animal 44 was 20.70 rads/s ± 5.26 (SD).

Angular velocity varied significantly (*p* < 0.05) with mass (*y* =  − 0.24 × + 1.01, *p* = 3.65 × 10^−2^, *t* =  − 2.16, *N* = 44, *R*^2^ = 0.10, statistical regression test). This correlation was not significantly different (*p* > 0.05) from the relationship predicted by hypothesis 3, *y* =  − 0.33 × + c, (*p* = 0.39, *t* = 0.86, *N* = 44, Statistical Regression test), which predicted constant angular energy density. Consequently, the angular velocity varied inversely with the length of the animal (L^−1^, or alternatively m^−0.33^), consistent with smaller locusts spinning faster such that the energy density of rotational kinetic energy is constant across all size locusts.

### Energetics of the jump

**Fig. 4:**
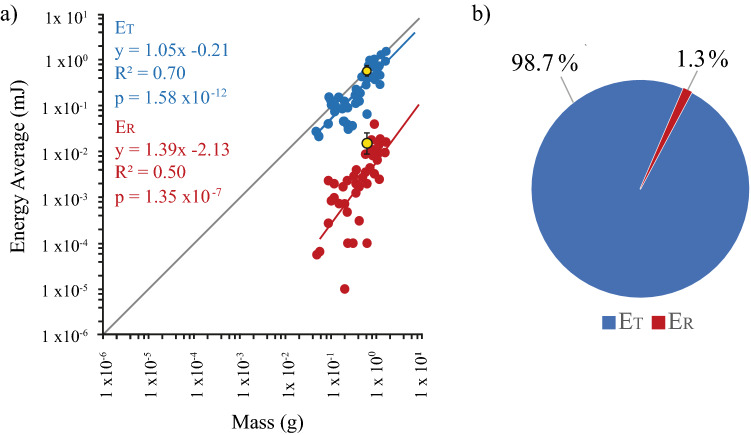
**a** Rotational kinetic energy (E_R_) and Translational kinetic energy (E_T_) of a locust’s jump, at take-off. Rotational kinetic energy (*p* = 1.35 × 10^−7^, *R*^2^ = 0.50) increases proportionally with translational kinetic energy (*p* = 1.58 × 10^−12^, *R*^2^ = 0.70). A line with a slope of *y* = 1x + c (shown in grey) is plotted to show the similarity in slope to our statistical trendlines (blue and red). **b** Energy budget of a locust’s jump, at take-off. On average, translational kinetic energy (including gravitational potential energy) accounts for 98.7% of the total energy and rotational kinetic energy accounts for 1.3% of the total energy budget, per jump

Because linear velocity was invariant with mass, translational kinetic energy (E_T_) (mJ) must increase proportionally to mass^1^. The average translational kinetic energy for the smallest locust (0.049 g) was 0.03 mJ. The average translational kinetic energy for the largest locust (1.50 g) was 1.56 mJ (Fig. 4a). Across 44 locusts, the average translational kinetic energy ranged from 0.02 to 1.56 mJ. The absolute maximum and minimum translational kinetic energy recorded across all jumps were 1.94 and 0.01 mJ. The average translational kinetic energy for animal 44 was 0.59 mJ ± 0.14 (SD).

Translational kinetic energy varied significantly (*p* < 0.05) with mass (*y* = 1.05 ×  − 0.21, *p* = 1.58 × 10^−12^, *t* = 9.89, *N* = 44, *R*^2^ = 0.70, Statistical Regression test). This correlation was not significantly different (*p* > 0.05) from *y* = 1x + c, (*p* = 0.62, *t* = 0.50, *N* = 44, Statistical Regression test), consequently translational kinetic energy increases proportionally to mass^1^.

Rotational kinetic energy (E_R_) (mJ) at take-off also proportionally increases with mass of the locust (Fig. 4a). The average rotational kinetic energy for the smallest locust (0.049 g) was 6.05 × 10^−5^ mJ. The average rotational kinetic energy for the largest locust (1.50 g) was 1.63 × 10^−2^ mJ. Across all 44 locusts the average rotational kinetic energy ranged from 1.01 × 10^–5^ to 4.18 × 10^−2^ mJ. The absolute maximum and minimum rotational kinetic energy recorded across all jumps were 1.09 × 10^−01^ and 3.44 × 10^−8^ mJ. The average rotational kinetic energy for animal 44 was 1.47 × 10^−2^ mJ ± 7.26 × 10^−3^.

Rotational kinetic energy varied significantly (*p* < 0.05) with mass (*y* = 1.39 ×  − 2.13, *p* = 1.35 × 10^−7^, *t* = 6.35, *N* = 44, *R*^2^ = 0.50, statistical regression test). This correlation was not statistically different (*p* > 0.05) from *y* = 1x + c (*p* = 0.08, *t* = 1.80, *N* = 44, statistical regression test), whereby rotational kinetic energy increases propotionally to mass^1^, or rather, rotational energy density remains constant (Hyp 3).

Translational kinetic energy and rotational kinetic energy both increased proportionally with mass (Fig. 4a). This allows us to calculate the distribution of energy in the energy budget that is put toward linear velocity and angular velocity, independent of the animal’s size.

For the 44 Locust, 98.7% + / − 0.2% of the energy budget, of a jump, goes toward linear velocity (Including gravitational potential energy), and 1.3% ± 0.1% of the energy goes toward rotation (pitch) (Fig. 4b).

### Intra-individual variation

**Fig. 5:**
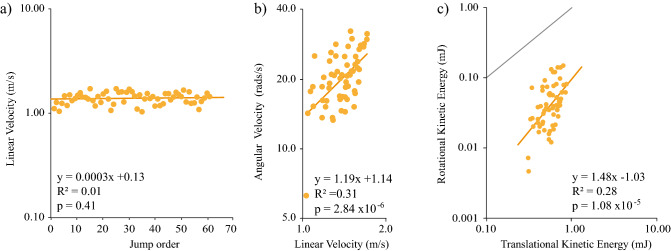
Intra-Individual variation **a** Linear velocity (m/s) of a locusts jump at take-off, over 61 consecutive jumps. Linear velocity does not vary with the number of consecutive jumps (*p* = 0.41, *R*^2^ = 0.01, Regression test). **b** Angular velocity (rads/s) and Linear velocity (m/s) of a locust’s jump at take-off, over 61 consecutive jumps. Angular velocity increases proportionally to linear velocity (*p* = 2.84 × 10^−6^, *R*^2^ = 0.31, Regression test). **c** Rotational kinetic energy (mJ) and Translational kinetic energy (mJ) of a locusts jump at take-off, over 61 consecutive jumps. Rotational kinetic energy increases proportionally to translational kinetic energy (*p* = 1.08 × 10^−5^, *R*^2^ = 0.28, Regression test). A line with the slope of *y* = 1 × + c (shown in Grey) is plotted to show the similarity in slope to that of our trendline (shown in black)

Linear velocity (m/s) at take-off, for locust number 44, does not vary with the number of consecutive jumps over time (Fig. 5a). The average linear velocity was 1.39 m/s ± 0.18 (S.D). The minimum and maximum linear velocities were 1.03 and 1.70 m/s respectively. Linear velocity showed no significant (*p* < 0.05) correlation over consecutive jumps (*y* = 0.0003 × + 0.13, *p* = 0.41, *t* = 0.82, *N* = 61, *R*^2^ = 0.01, Statistical Regression test).

Angular velocity (rads/s) at take-off (Fig. 5b) varied significantly (*p* < 0.05) with linear velocity (*y* = 1.19 ×  + 1.14, *p* = 2.84 × 10^−6^, *t* = 5.17, *N* = 61, *R*^2^ = 0.31, statistical regression test). This correlation was not significantly different (*p* > 0.05) from *y* = 1 × + c (*p* = 0.42, *t* = 0.82, *N* = 61, statistical regression test), whereby fast jumps spin faster.

Finally, rotational kinetic energy (mJ) (Fig. 5c) significantly varied (*p* < 0.05) with translational kinetic energy (mJ) (*y* = 1.48 ×  − 1.03, *p* = 1.08^−5^, *t* = 4.81, *N* = 61, *R*^2^ = 0.28, statistical regression test). This correlation was not significantly from *y* = 1x + c (*p* = 0.12, *t* = 1.56, *N* = 61, statistical regression test). Therefore, rotational kinetic energy and translational kinetic energy increase proportionally.

## Discussion

How is angular rotation affected by the mass of a locust? To answer this question, we measured linear velocity and angular velocity at take-off for differently sized locusts. As expected, the linear velocity was independent of mass and translational kinetic energy was proportional to mass, both of which are consistent with typical LaMSA movements (Ilton et al. 2018; Longo et al. 2019; Sutton et al. 2019). We observed that the locust’s angular rotation (pitch) was proportional to mass^−0.33^ (Fig. 3) i.e. smaller jumpers rotate faster. This effect was consistent with a conservation of angular energy density across all masses. Rotational kinetic energy therefore proportionally increased with mass^1.0^ (Fig. 4). There was a lot of variation in the angular velocity (*R*^2^ = 0.10), consistent with the observation that pitch rotation can be quite variable for locusts (Gvirsman et al. 2016). The observed relationship between mass and angular velocity ($\dot{\theta }$ is proportional to mass^−0.24^) was statistically not distinguishable from our hypothesised relationship ($\dot{\theta }$ is proportional to mass^−0.33^).

The observed relationship between mass and rotational kinetic energy ($\frac{1}{2}I{\dot{\theta }}^{2}$ is proportional to mass^1.39^) was also statistically not distinguishable from our hypothesised relationship ($\frac{1}{2}I{\dot{\theta }}^{2}$ is proportional to mass^1.0^), and there was much less scatter in this relationship (*R*^2^ = 0.50), indicating that the energy density in rotation was constant. On average, locusts dedicate 1.3% of their total jump energy budget to angular rotation, and this is independent of size (Fig. 4). This small proportion of the energy budget allows the locust to generate dozens of radians/secs of rotation rate without angular rotation greatly affecting the total energy budget of a jump.

The proportional linear correlation found between linear velocity and angular velocity, supports the idea that locusts do not independently control pitch and linear speed. The total amount of energy put into the system can be varied by simply storing more or less energy in the elastic processes of the leg, but, once the spring recoils, the distribution of energy allocated to linear and rotational velocity appears fixed. If locusts are not able to control their angular velocity, then their ability to land feet first will be subjective to the distance of a target. Slow motion footage of the locusts in this study frequently saw locusts reach the target but land at the wrong angle. Imprecise landing has also been observed for locusts jumping to flat horizontal surfaces (Faisal and Matheson 2001). We cannot determine if the locusts ‘choose’ not to control their pitch rate to land squarely on the target or if they were unable to do so. However, if locusts are not able to control their angular rotation, then it should be possible to calculate the distances at which a locust of known mass is not able to land feet first, based on its take-off velocity.

The smaller the locust the faster it spun. This is because the fixed 1.3–98.7% partition between rotational and linear translational kinetic energy caused smaller locusts to spin faster. This leads to the hypothesis that smaller insects, in general, have a more difficult time jumping at lower rotation rates (i.e., the smaller you are the faster you spin). A review of the literature (Table 1) suggests that this may be so, with the highest rotation rates being found in the smallest insects. A notable exception is the snow flea (*Boreus:* Burrows 2011), which despite its 2.9 mg mass has a rotation rate (2 Hz) comparable to a 1.5 g locust (*Schistocerca gregaria*) (1.7 Hz). Unlike the other similarly sized insects however, *Boreus* uses four legs to jump instead of two. The four jumping legs of *Boreus* may give it a stable platform from which to jump, thus allowing incredibly low rotation rates, despite its small size.

**Table 1:** Angular rotation (Pitch) at take-off, from a selection of jumping insects from literature

Species	Mass (mg)	Body length (mm)	Angular rotation at take-off (Pitch) (rad s^−1^)	Angular rotation (Hz)
*Cacopsylla peregrina* (Burrows 2012)	0.7	1.9	2100.0	340.0
*Archaeopsylla erinacei* (Sutton and Burrows 2011)	0.7	1.8	110.0	17.0
*Psyllopsis fraxini* (Burrows 2012)	1.2	2.2	1300.0	200.0
*Chaetocnema aridula* (Nadein and Betz 2016)	1.4	2.1	980.0	160.0
*Psylla alni* (Burrows 2012)	2.8	4.0	1400.0	220.0
*Boreus* (Burrows 2011)	2.9	3.4	13.0	2.0
*Apthona cyparissiae* (Nadein and Betz 2016)	4.4	3.5	370.0	58.0
*Xya capensis* (Burrows and Picker 2010)	8.3	5.6	200.0	190.0
*Odontomachus bauri* (Patek et al. 2006)	11.9	13.4	390.0	63.0
*Philaenus spumarius* (Linnaeus) (Burrows 2003)	12.3	6.1	500.0	80.0
*Issus coleoptratus* (Burrows 2009)	21.5	6.7	270.0	43.0
*Stagmomantis theophila (5*^*th*^* Instar)* (Sutton et al. 2016)	85.7	30.6	21.0	3.3
*Lanelater judaicus* (Ribak and Weihs 2011)	200.0	20.3	31.0	5.0
*Schistocerca gregaria (Presented here)*	1500.0	47.0	11.0	1.7
* (*Cofer et al. 2010*)*			13.0	2.1
*Prosarthria teretrirostris (female)* (Burrows and Wolf 2002)	1540.0	104.4	52.0	8.3

Where not explicitly stated in the paper, rotation rates were taken by analysing supplementary videos from the manuscripts, using Tracker. Once in flight, angular rotation can greatly vary due to air resistance and other factors such as the use of wings, but we did not measure these effects. This Table is ordered by body mass from smallest to largest

Muscle driven jumpers, such as praying mantises, are able to control their angular rotation to aid landing by counter rotating their legs and abdomen to adjust their pitch (Burrows et al. 2015). Unlike the mantises, locusts have relatively small front legs of very little mass, which likely prevents them from counteracting their rotation in the same way. Locusts are, however, able to influence their angular rotation to bias a head-up tail-down direction through contraction of the dorsolongitudinal muscle to flex their abdomen (Cofer et al. 2010). Abdominal control of angular rotation in locusts is limited to influencing the direction of rotation only. This technique does not allow locusts to fine tune their rotation to the exact angle of pitch required to land on a surface feet first.

The velocity at which locusts jump may provide too little time for neural control of their pitch. However, it would presumably be very easy for the locust to evolve a mechanism to control pitch or alter their jumping behaviour, such as moving the position of their feet (and thus their thrust vector) relative to their centre of mass, prior to take-off (Cofer et al. 2010). Therefore, we must consider why pitch is not controlled and its possible benefits.

It has been suggested that locusts might benefit from fast angular rotation during take-off for flight initiation and escape jumps, used when presented by a threat (Gvirsman et al. 2016). Fast angular velocity could be used to confuse a predator, making it difficult to track the position and direction of the fleeing insect (Burrows 2011). However, this would only be effective to escape an immediate strike from the predator, as uncontrolled angular velocity hinders the locust’s ability to land up-right, greatly increasing the reset time between consecutive jumps and thus decreases the likelihood of escaping pursuit. Alternatively, the spin may stabilise the locust from perturbation in the air, similar to the rifling of a bullet (Baranowski 2013). But we do not have the data to test this hypothesis. Which once again leaves the question of why don’t locusts consistently control their angular velocity?

In conclusion, locust’s angular energy density remains constant causing angular rotation to be proportional to mass^−0.33^, or alternatively the angular velocity is inversely proportional to the animal’s length. The energy budget per jump is fixed and on average locusts dedicate 1.3% of this budget to angular rotation. It may be a general principle that small biological LaMSA jumpers are unable to independently vary take-off velocity and rotation rate.

## Supplementary Information

Below is the link to the electronic supplementary material.

Supplementary movie 1 The jump of a 1st instar locust (0.09 g) and 6th instar locust (0.82 g) both filmed at 1000 frames/s. At take-off the 1st instar locust is spinning with more than four times the angular velocity of the larger locust (60.41 and 12.97 rads/s respectively). While in the air the rotation of both insects decelerated (MP4 1370 KB)

## Data Availability

Data collected in this study is available on the BioStudies online database, accession number S-BSST1003. Alternatively, data is available on request from the authors.
